# Comment on “Advancing material property prediction: using physics-informed machine learning models for viscosity”

**DOI:** 10.1186/s13321-025-01070-9

**Published:** 2025-08-28

**Authors:** Maximilian Fleck, Samir Darouich, Marcelle B. M. Spera, Niels Hansen

**Affiliations:** 1 Institute of Thermodynamics and Thermal Process Engineering, University of Stuttgart, Pfaffenwaldring 9, 70569 Stuttgart, Germany; 2 Institute for Artificial Intelligence, University of Stuttgart, Universitätsstraße 32, 70569 Stuttgart, Germany; 3 Institute for Theoretical Chemistry, University of Stuttgart, Pfaffenwaldring 55, 70569 Stuttgart, Germany

**Keywords:** Eyring rate theory, Viscosity, Physics-inspired neural network

## Abstract

When data availability is limited, the prediction of properties through purely data-driven machine learning (ML) is challenging. Integrating physically-based modeling techniques into ML methods may lead to better performance. In a recent work by Chew et al. (“*Advancing material property prediction: using physics-informed machine learning models for viscosity*”) descriptors from classical molecular dynamics (MD) simulations were included into a quantitative structure–property relationship to accurately predict temperature-dependent viscosity of pure liquids. Through feature importance analysis, the authors found that heat of vaporization was the most relevant descriptor for the prediction of viscosity. In this comment, we would like to discuss the physical origin of this finding by referring to Eyring’s rate theory, and develop an alternative modeling approach using a thermodynamic-based architecture that requires less input data.

**Supplementary Information:**

The online version contains supplementary material available at 10.1186/s13321-025-01070-9.

## Introduction

Recently, Chew et al. [1] described the quantitative structure–property relationship (QSPR) of battery-relevant solvents using physics-informed machine learning (ML) [2–4]. Best performing models were a descriptor-based light gradient-boosting machine (LGBM) model [5] and a graph neural network (GNN) model using edge pooling [6, 7] and jumping knowledge [8]. Regardless of descriptor-based or graph-based models, the inclusion of molecular dynamics (MD) descriptors representing intermolecular interactions was found to be beneficial, in agreement with previous works targeting solvation free energies [9]. Chew et al. [1] found the heat of vaporization to be the most useful descriptor relevant to viscosity even in the presence of hundreds of descriptors. In this comment, we would like to add to the original article by further discussing the relation between viscosity and energy of vaporization.

In 1937, Ewell and Eyring [10] developed a model to describe viscosity based on a reaction rate theory. The authors considered the viscous flow as a chemical reaction in which the event is the passing from one equilibrium position to another over a potential energy barrier. For low to moderate pressures, the authors correlate the viscosity to a function of temperature, molar volume, and energy of vaporization [10–12]:

1 $$\begin{aligned} \eta&= 1.090 \cdot 10^{-3} \frac{M^{\frac{1}{2}} T^{\frac{3}{2}}}{V^{\frac{2}{3}} \Delta E_{\text {vap}}} \exp \bigg ( \frac{\Delta E_{\text {vap}}}{nRT}\bigg ) \end{aligned}$$

with energy of vaporization $\Delta E_{\text {vap}}$, molar volume *V*, molar mass *M*, temperature *T*, and ideal gas constant *R*. The constant *n* relates the activation energy for viscous flow as some fraction of the energy of vaporization, with $n=3$ for spherical molecules and $n=4$ for molecules not of spherical symmetry. Therefore, the activation energy of flow is directly related to the energy of vaporization. For elongated molecules, preferred orientations are possible and a smaller fraction of the energy of vaporization is sufficient to activate flow. This leads to the useful concept that viscous flow (and consequently molecular transport phenomena in general) can be described as vaporization in one degree of freedom. Ewell and Eyring [10] show in their work that, even though systematic deviations are present, the qualitative behavior of the viscosities can be predicted based on vaporization energies.

## Methods

Equation 1 was extended by an additional parameter $a_0$ to account for systematic deviations and written in logarithm form,

2 $$\begin{aligned} \ln (\eta )&= \ln \bigg ( 1.090 \cdot 10^{-3} \frac{M^{\frac{1}{2}} T^{\frac{3}{2}}}{V^{\frac{2}{3}} \Delta E_{\text {vap}}} \bigg ) + \frac{\Delta E_{\text {vap}}}{nRT} + a_{0} \end{aligned}$$

**Fig. 1:**
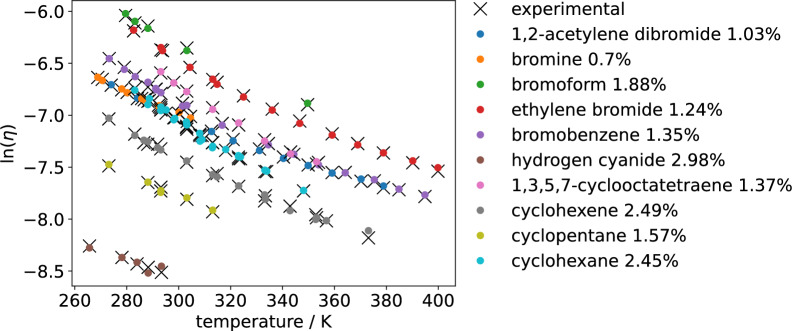
Viscosity model from Eq. 2, with *n* and $a_0$ fitted to experimental data. Results are shown for the first 10 species of the dataset. The deviations are given as mean absolute relative deviations in percent. The temperature dependence is modeled very well by the Eyring relation and the MD descriptors

Equation 2 was tested with the viscosity dataset provided by Chew et al. [1], fitting the parameters *n* and $a_0$ for every species to match experimental data. The dataset contains 957 species and 3582 data points. $\Delta E_{\text {vap}}$, *V*, and *T* are available in the dataset as MD descriptors, with $\Delta E_{\text {vap}}$ calculated as $\Delta H_{\text {vap}} - \Delta (pV)$. We assumed the gaseous phase to be an ideal gas. Results are shown in Fig. 1 for selected species from the dataset, all with more than four experimental measurements available for viscosities.

**Fig. 2:**
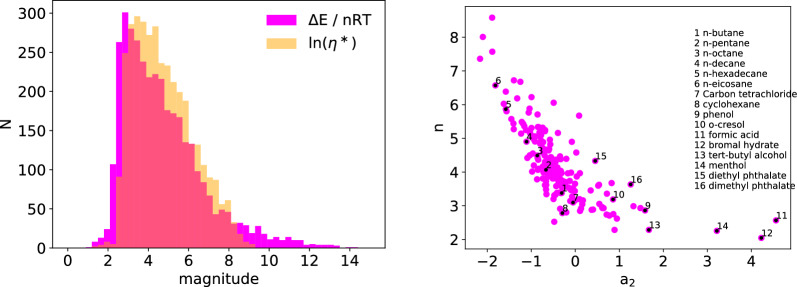
$\ln (\eta ^*)$ and ${\Delta E}/{nRT}$, with $n=4$, showing similar behavior and distribution after their magnitudes have been shifted in the same range using the parameter $a_1$ (left). Relation between *n* and $a_2$ showing an almost linear relationship between the parameters for alkanes and that n indeed represents the chain-length

Since the qualitative behavior of viscosity with temperature is captured well by the Eyring approach, a predictive model was developed. For a neural network architecture, we define $\eta ^*$ as follows:

3 $$\begin{aligned} \ln (\eta ^*)&= \ln (\eta ) - \ln \bigg ( 1.090 \cdot 10^{-3} \frac{M^{\frac{1}{2}} T^{\frac{3}{2}}}{V^{\frac{2}{3}} \Delta E_{\text {vap}}} \bigg ) + a_1 \end{aligned}$$

with $a_1$ a constant defined as $a_0 = a_2 - a_1$, which is used to shift the mean values of the known parts of Eq. 3 to the same origin. Thus, the model can be formulated as follows

4 $$\begin{aligned} \ln (\eta ^*)&= \frac{\Delta E_{\text {vap}}}{nRT} (n_0+1) + a_2 \end{aligned}$$

with unknown parameters $n_0$ and $a_2$. As a starting point, we assumed the arbitrary species in the dataset to be chain-shaped, therefore we use $n = 4$ [10]. The graph neural network is trained to predict $n_0$ and $a_2$, and consequently $\ln (\eta ^*)$, with ${\Delta E}/{nRT}$ being a model input. Figure 2 shows that $\ln (\eta ^*)$ and ${\Delta E}/{nRT}$ are closely related. This indicates that it is very promising to learn $\ln (\eta ^*)$ based on ${\Delta E}/{nRT}$ when they are coupled through the Eyring relation.

**Fig. 3:**
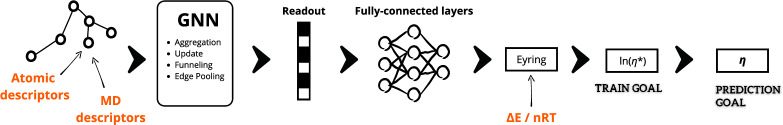
Model overview with model inputs shown in orange. The output of the fully-connected layers, $n_0$ and $a_2$, are the other inputs of Eq. 4. Viscosity $\eta$ is then predicted using Eq. 3

The model is shown in Fig. 3 and consists of a graph neural network with node features, followed by a fully connected network and the Eyring relation (Eq. 4). The graph neural network uses the following 9 atomic descriptors for models trained without MD descriptors: Lennard–Jones/Coulomb parameters $\sigma$, $\epsilon$, and *q* [13, 14] for a set of 45 atom types defined based on the species in the data set, the number of bonds and number of hydrogen atoms bound to each heavy atom, the (united) atom mass including bound hydrogen atoms, the ring size (0 if not in ring), a charge map encoding if a partial atomic charge is positive, negative or neutral and the atom number in the periodic table.

By contrast, for our model with MD descriptors we only used the atom number in the periodic table as atomic descriptor. The atomic descriptors used in this work could be considered of lower quality compared to the 75 DeepAutoQSAR features. Nevertheless, the model should remain competitive because we build a physics-inspired architecture with the Eyring relation. The MD descriptors used are the ones provided by Chew et al. [1]. We want to highlight that our model is only indirectly informed about the temperature through the Eyring relation and the MD descriptors.

We tested graph neural networks with different message passing schemes [15–17]. For readout, we used edge pooling [6, 7], jumping knowledge [8] with concatenation, combined with different pooling types [16, 18, 19]. Pooling types and message passing scheme are degrees of freedom of the hyperparameter optimization. The number of nodes, depth of the graph neural network and the fully connected layers, and if they are funneled over depth or not, are also hyperparameters. We used an out-of-sampling approach splitting along species [1], getting train, validation, test splits of 0.70, 0.15, 0.15 relative to the total number of data points, with early stopping for training. During training we used dropout. Hyperparameters are tuned via random search. Hyperparameters of the ML model are reported in Additional file 1: Table S1.

## Results

**Fig. 4:**
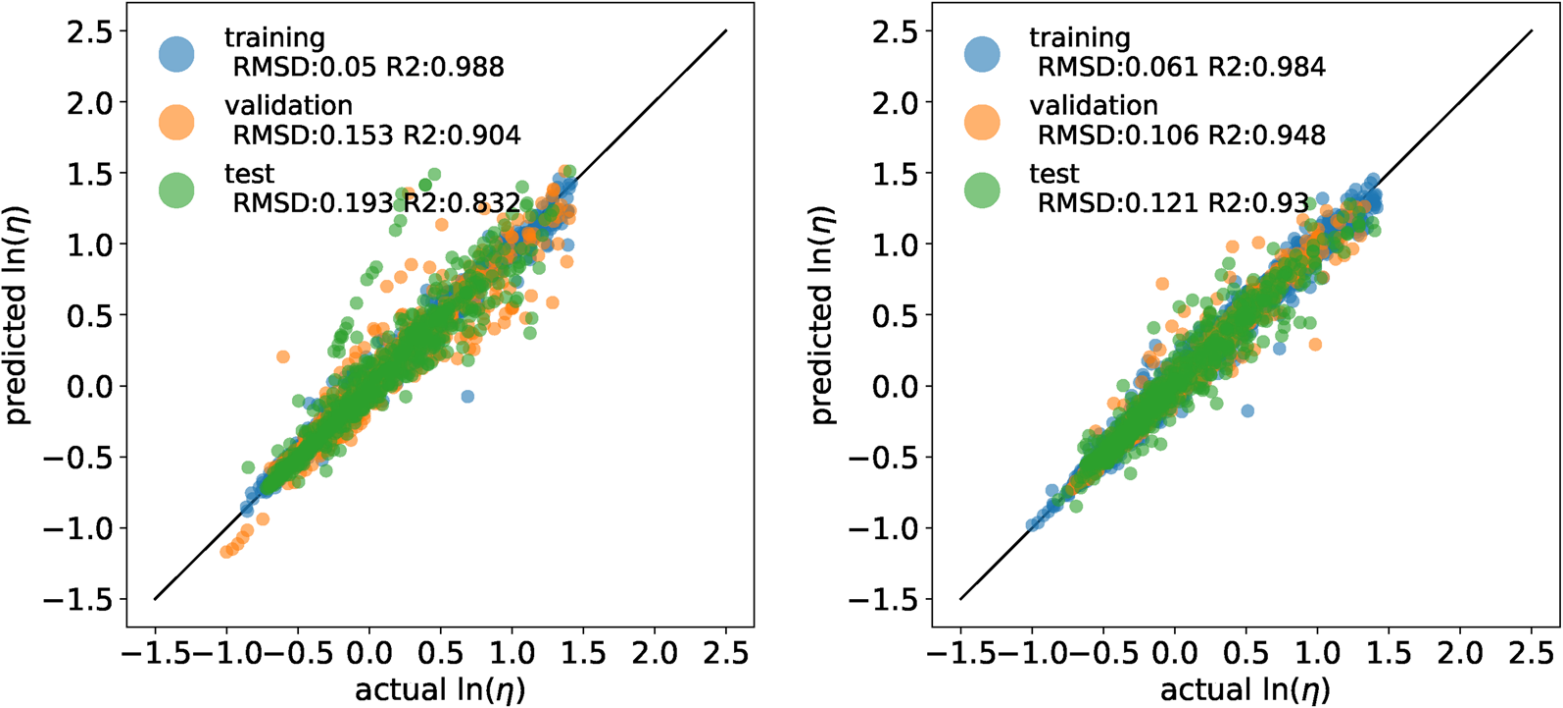
Results of a model trained without (left) and with MD descriptors (right). We used the same scale as Chew et al. [1] for comparability

Figure 4 shows that our model has promising capabilities. The model without MD features has very small training RMSD but fails to extrapolate to some species outside training data. As proposed by Chew et al. [1], using MD descriptors improves the generalization capabilities of our model. The dependency on the specific train/validation/test split also corresponds to the results of Chew et al. [1]. We also evaluated a model without including the Eyring equation, showing less good performance (see Additional file 1: Fig. S1). Moreover, the influence of the training data set size was studied, indicating that inclusion of the Eyring equation decreases the required training data set size (see Additional file 1: Figs S2 and  S3).

## Conclusion

In this comment, we discussed why the heat of vaporization serves as significant descriptor relevant to viscosity (and, generally, transport phenomena) using the Eyring rate theory. We showed that this theory has great predictive capabilities for viscosities when driven by MD features. We implemented a physically-inspired architecture containing Eyring’s theory which worked very well to predict viscosites. We also confirmed the findings of Chew et al. [1] that molecular simulations can deliver powerful descriptors and improved prediction accuracy. We note that the model proposed in the present work requires less training data to achieve comparable prediction quality as the model of Chew et al. [1]. The curated viscosity dataset from Chew et al. [1] allows for reproducibility of the results and the development of further methods. In future work the approach can be combined with DeepAutoQSAR [20], or descriptors based on $\sigma$-profiles [21]. An extension to mixtures could be based in modeling the excess Gibbs energy [22].

## Supplementary Information

**Additional file 1.**

## Data Availability

The code and a copy of the original dataset from Chew et al. are available on github: https://github.com/maxfleck/comment-deep-eyring-architecture.

## Abbreviations

GNN: Graph neural network
LGBM: Light gradient-boosting machine
MD: Molecular dynamics simulations
ML: Machine learning
QSPR: Quantitative structure–property relationship
RMSD: Root mean square deviation
vap: Vaporization
