# Supplementary material for: Comment on “Advancing material property prediction: using physics-informed machine learning models for viscosity”
Source: J Cheminform. 2025 Aug 28;17:131. doi: 10.1186/s13321-025-01070-9 (PMC12392590; doi:10.1186/s13321-025-01070-9)
Supplement: Supplementary file 1 — Additional file 1. [file 13321_2025_1070_MOESM1_ESM.pdf]

Additional file
Comment on "Advancing material property prediction: using physics-informed machine learning models for viscosity"

Maximilian Fleck[1], Samir Darouich[2,3], Marcelle B M Spera[1] and Niels Hansen[1]

[1] *Institute of Thermodynamics and Thermal Process Engineering*, University of Stuttgart,Pfaffenwaldring 9, Stuttgart, 70569, Germany
[2] *Institute for Artificial Intelligence*, University of Stuttgart, Universitätsstraße 32, Stuttgart, 70569, Germany
[3] *Institute for Theoretical Chemistry*, University of Stuttgart, Pfaffenwaldring 55, Stuttgart, 70569, Germany

maxi_fleck@posteo.com, hansen@itt.uni-stuttgart.de

Table S1: Details of the machine learning method.

learning rate	0.001
batch size	32
number of atomic features	1
graph layers/convolutions	12
graph hidden features	124
graph readout frequency	2
network hidden layers	3
network hidden nodes	50
aggregation	mean
funnel graph	false
funnel network	false
graph convolution	SAGEConv
pooling	addmax

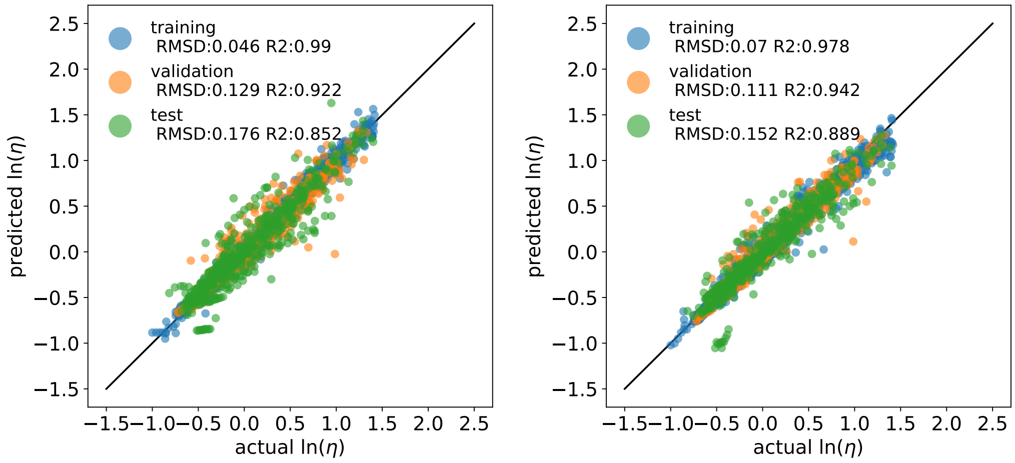

Figure S1: Results of two models similar to the ones of Chew et al. (without the Eyring equation included) trained on the same train/val/test split and same hyperparameters than Fig. 4 (right) from the main text. The model on the left was trained with 9 atomic descriptors in addition to the MD features. The model on the right only with 1 atomic descriptor in addition to the MD features. The model using more atomic descriptors shows more overfitting on the train set and worse extrapolation to the test set. We want to point out that both models perform comparably to the model of Chew et. al. using 75 atomic descriptors. This questions the need for dedicated atomic descriptors when MD features are available.

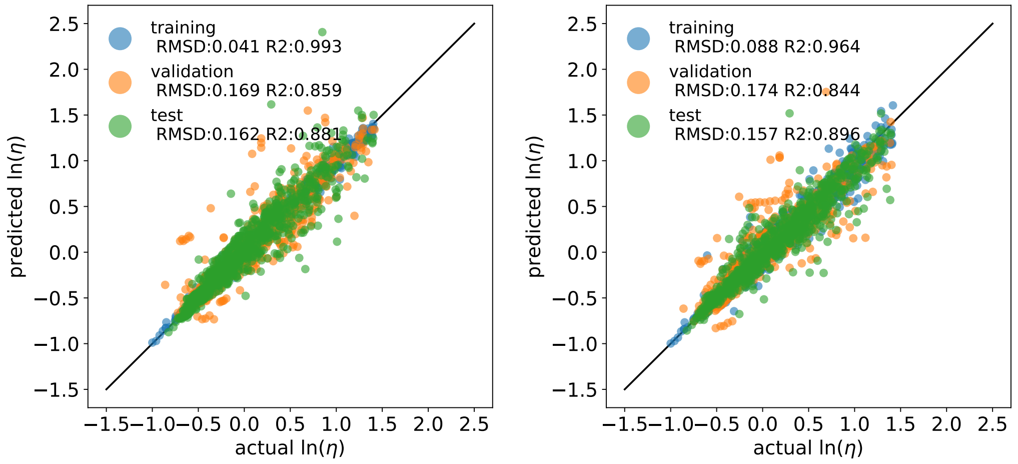

Figure S2: Results of two training runs of the Eyring model using MD descriptors, i.e., trained on two different 50/25/25 train/val/test splits and same hyperparameters than Fig. 4 (right) from the main text. The model was trained with roughly half of the data compared to the best model of Chew et. al. (1788 vs. 3500) whilst showing comparable performance highlighting the capabilities of the Eyring model.

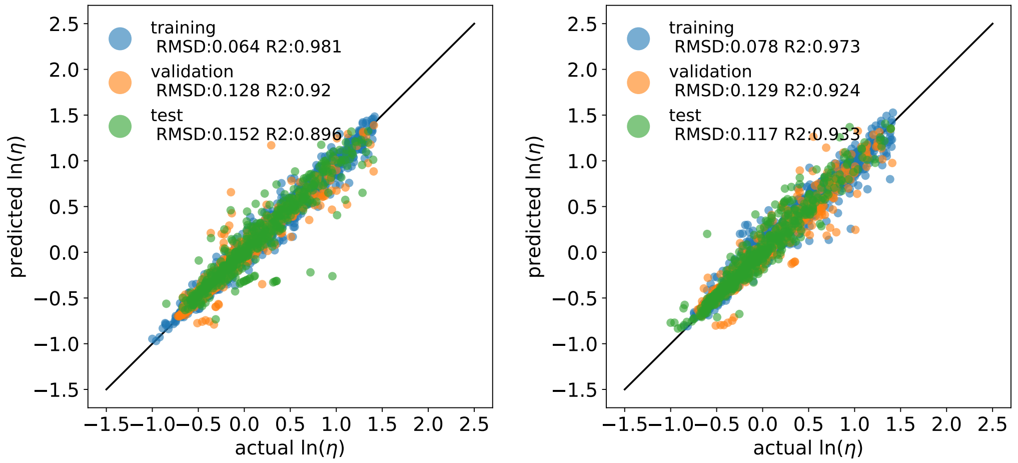

Figure S3: Results of two training runs of the Eyring model using MD descriptors, i.e., trained on two different 70/15/15 train/val/test splits. The models form the lower and upper ends in the metrics obtained with the Eyring GNN model type. It is also noticeable here that the models that extrapolate very well in the test set perform slightly worse in the training set, i.e., they do not overfit. The use of fewer atomic features seems to help prevent overfitting.
